# Modelling the emergence of cities and urban patterning using coupled integro-differential equations

**DOI:** 10.1098/rsif.2022.0176

**Published:** 2022-05-04

**Authors:** Timothy D. Whiteley, Daniele Avitabile, Peer-Olaf Siebers, Darren Robinson, Markus R. Owen

**Affiliations:** ^1^ School of Mathematical Sciences, University of Nottingham, Nottingham, UK; ^2^ School of Computer Science, University of Nottingham, Nottingham, UK; ^3^ School of Architecture, University of Sheffield, Sheffield, UK; ^4^ Department of Mathematics, Vrije Universiteit Amsterdam, Amsterdam, The Netherlands

**Keywords:** urban, ‌pattern formation, length scale, mathematical modelling, differential equation, stability analysis

## Abstract

Human residential population distributions show patterns of higher density clustering around local services such as shops and places of employment, displaying characteristic length scales; Fourier transforms and spatial autocorrelation show the length scale between UK cities is around 45 km. We use integro-differential equations to model the spatio-temporal dynamics of population and service density under the assumption that they benefit from spatial proximity, captured via spatial weight kernels. The system tends towards a well-mixed homogeneous state or a spatial pattern. Linear stability analysis around the homogeneous steady state predicts a modelled length-scale consistent with that observed in the data. Moreover, we show that spatial instability occurs only for perturbations with a sufficiently long wavelength and only where there is a sufficiently strong dependence of service potential on population density. Within urban centres, competition for space may cause services and population to be out of phase with one another, occupying separate parcels of land. By introducing competition, along with a preference for population to be located near, but not too near, to high service density areas, secondary out-of-phase patterns occur within the model, at a higher density and with a shorter length scale than in phase patterning. Thus, we show that a small set of core behavioural ingredients can generate aggregations of populations and services, and pattern formation within cities, with length scales consistent with real-world data. The analysis and results are valid across a wide range of parameter values and functional forms in the model.

## 1. Introduction

The world is becoming increasingly urban. In 2007, the global urban population overtook the rural and, by 2050, two-thirds of the world population is expected to live in cities [1]. Cities are vitally important as hubs of business, commerce, social interaction and all the other necessary services that help us to survive. They are highly complex, resource consuming and self-organizing systems, as people are glued together by the services that support them but also pushed away by the problems that densification causes.

Issues of urban density will affect transport networks, vehicle kilometres travelled [2], public transport feasibility [3] as well as social implications such as quality of life [4].

The existence and size of cities is a phenomenon largely driven from the bottom up, by the choices of individuals and firms. Yet mathematical patterns persist such as Zipf’s Law [5,6] which states that within a country or region, a city’s size is inversely proportional to its rank within that region.

**Figure 1.**
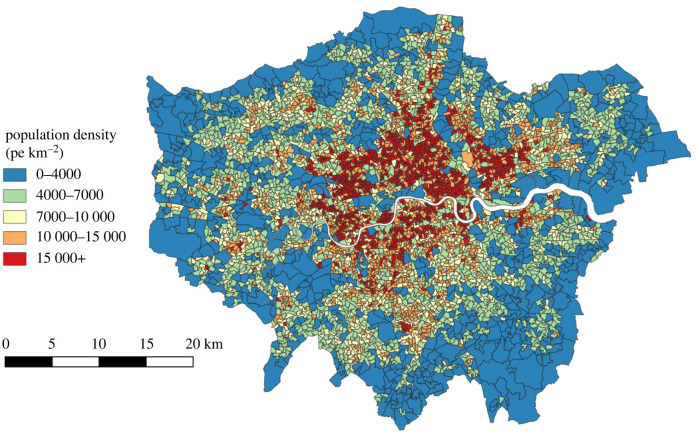
Population density distribution in London from 2019 Population data [7], illustrating decreasing density overall with distance from the centre, with accompanying patchiness. Central London in particular displays a ‘density crater’ with lower population density due to the competing presence of commercial land use. Other patches are driven by commercial competition and other non-residential land use such as greenspace and parks.

As a motivating example of population density within a UK city, we show a map of London in figure 1. This is an old city which has grown and absorbed many smaller towns around it over time. There is a clear increase in population towards the city centre, and a notable population crater in the middle where services dominate. There are further areas of patchy residential patterning seen within the city as land uses such as parks, retail and industry outcompete population in certain areas.

There is a global imperative to better understand how cities can be structured to function in more efficient and environmentally benign ways. To this end, we consider here the emergence of patterns of population density; the arrangement of people into and within cities. A mathematical model that supports emergent cities and subsequent secondary patterning within the city may deepen our insights into how cities’ spatial structures emerge and, more importantly, reveal how these processes might be influenced.

In this paper, we will model and analyse the emergence of cities using an integro-differential equation approach, assuming a preference for people and services to locate near to each other, with distance dependence encapsulated in spatial weight kernels. Firstly, in §2, we will look at precedent models showing the value of differential equation-based modelling of cities and highlighting the scope for further work. In §3, we show how population density in parts of the UK demonstrates emergent length scales of 45−50 km between cities and, in appendix A, we show a length scale of 200 km between cities in the USA. This analysis provides the motivation for a new, explanatory, model developed in §4, which explains the emergence of population patterns. Linear stability analysis around the homogeneous steady state, a technique not yet applied in the urban modelling literature, is used to predict emergent length scales from the model in §5. This model is developed further in §6 to show areas of within city structure, as population and services develop out-of-phase patterns. Lastly, in §7, we add population growth to the model to show how cities may grow and agglomerate, developing structure as they do. We conclude with a discussion of our main findings and directions for future work in §8.

## 2. Mathematical models for urban population density

In 1951, Clark [8] proposed the empirical model that, excluding the central business district (CBD), population density in cities declines exponentially with distance from the centre. Subsequently, Newling [9] suggested a revised quadratic exponential empirical model which captures the low central population density that corresponds to a city’s CBD. Bertaud [10] shows examples of nine world cities that display this characteristic profile. Newling postulated that as the population grows the parameters change to create and reinforce this central dip.

One key model of polycentric configurations and the interaction between households and firms is due to Fujita & Ogawa [11]. Their economic agglomeration-based model adapts Alonso’s well-known 1960s bid rent theory [12] where retail, manufacturing and residents compete for land; each having maximum bids for a given distance from the CBD. In Fujita and Ogawa’s model, land is occupied by population and firms. Households wish to maximize the commodities that they can gain from firms by balancing income, rent and travel costs while firms wish to maximize their profit by balancing the value gained by locating close to other businesses (captured by a weighted integral) against wage and rent costs. Hypothetical city structures are set up, and the parameters under which these are valid are analysed to explain the possible equilibrium states of the system. They show that there may be both continuous and sudden structural changes in the city dynamic at the boundaries of where an equilibrium is supportable.

In one of the first dynamic spatio-temporal models, Bracken & Tuckwell [13] used an integro-differential equation for population in one radial dimension. Their model has three terms: diffusion of population, logistic growth and an integral term that represents growth inhibition at distance *r* from the city centre. This integral is proportional to the total population between the city centre and *r*, emulating the negative impacts of travel congestion and increased house prices.

A more detailed and dynamical model of the growth of urban centres in a larger region was developed by Allen & Sanglier [14], building on their earlier work [15]. They propose a model with logistic growth of population density at a set of discrete locations, with a carrying capacity at each location which depends on jobs of different types, and with migration from higher to lower densities (penalized by distance moved). This model shows how interacting dynamics of population and jobs or services can produce centres of attraction; the resulting patterns always develop with population and jobs co-located in a self-reinforcing pattern.

Alan Wilson’s entropy maximization [16] is a technique from transport modelling that has been adapted to model shopping power per location, considering monetary flows to predict sites with greatest potential for service growth. The transition from small corner shop to large supermarkets [17] is explained by modelling the advantage of larger floor area compared to the travel costs to such sites. Fry & Smith [18] recently extended this approach to develop a time-dependent model; they use entropy to define the profit of a configuration and hence drive growth of each retail location. Simplifications of their model allow asymptotic analysis on customer preference towards larger floor areas, showing a bifurcation from a homogeneous state where there are no differences between centre sizes, to a ‘winner takes all’ dynamic, whereby the centre with the original maximum size is the site that dominates the market.

Lastly, a number of recent papers on a model of reaction–diffusion equations of population and wealth distribution have been released [19–21]. In their statistical analysis of the population landscape [19], the authors show spatial correlation across Canada, Australia and Mongolia that cannot be explained by environmental factors alone, highlighting the need for explanatory modelling. In all three papers, they model population wealth growth. Low and high incomes both give rise to lower growth of population, whereas growth in wealth increases with both increased wealth and increased population. In the non-spatial system, there are multiple steady states with complex bifurcations leading to sudden boom or collapse in the economy or population levels. In multiple dimensions, their stability analysis shows the emergence of characteristic length scales.

Since the 1980s, there has been a growth of bottom-up computational approaches to urban population modelling, without a corresponding development of mathematical theory to uncover general principles. Cellular automata [22–24] and agent-based models [25–30] are powerful tools for simulating urban populations and for making data-driven predictions about the future state of a city, but they tend to lack explanatory power. On the other hand, parsimonious models of urban populations can show how the overall shape of a city may form [9,13], how multiple centres can dynamically emerge [15] and how to identify such transitions between equilibria [11]. In this paper, we uncover a set of underlying principles that can drive city formation and patterning, based on spatial kernels capturing distance preferences. We focus on the appearance of characteristic length scales and the emergence of complementary patterns, which is novel in the urban literature.

## 3. Length scales between cities

We consider two methods to quantify the length scales between cities: two-dimensional spatial auto-correlation and the Fourier transform of a one-dimensional transect. These techniques are applied to UK Office for National Statistics (ONS) mid-2016 population density data for lower layer super output areas in England and Wales (LSOAs, average 1700 people) [31].

Spatial autocorrelation is computed by comparing Moran’s I at a set of distances [32]. Moran’s I is a measure from −1 to 1 of the correlation of points separated by distance *d*. We group the set of pairs of LSOAs into those with centroid distance 0–1 km, 1–2 km, etc., and calculate the correlation of points in these groups.

**Figure 2.**
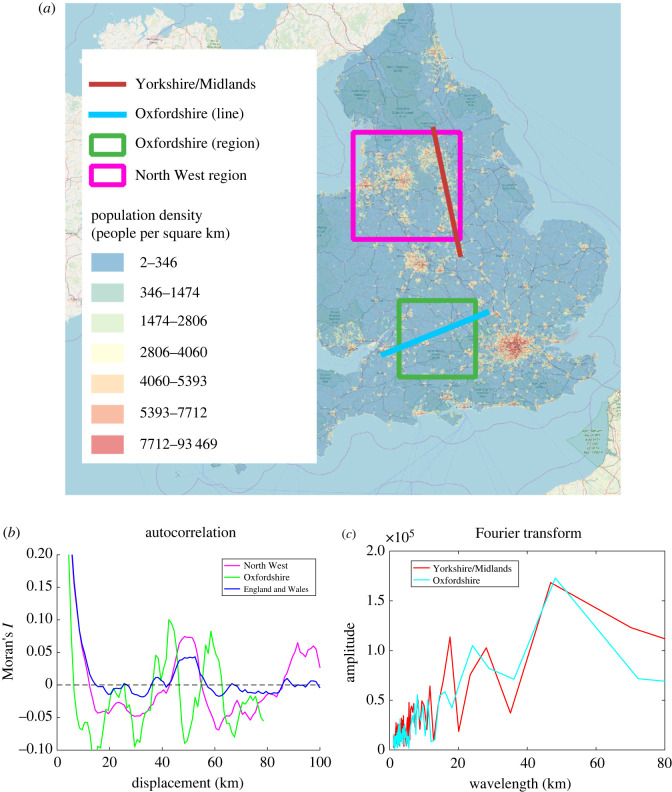
(*a*) A map of the population density of the UK showing the regions and lines analysed in this section. Background mapping © Open street map contributors. (*b*) Autocorrelation for England and Wales, the North West and Oxfordshire regions showing a dominant length scale of 50 km and 43 km, respectively. (*c*) Fourier transform of the Yorkshire/Midlands and Oxfordshire lines both showing a characteristic length scale of 45–50 km.

We calculate *I*(*k*) for the whole of England and Wales (encompassing 25 053 LSOAs), as well as showing the smaller regions of: the North West, including Manchester, Liverpool, Leeds, Sheffield and Nottingham (6712 LSOAs); and the region of Oxfordshire including Oxford, Swindon and Reading (1549 LSOAs) as can be seen on the map in figure 2.

Secondly, we take a Fourier transform of a transect through the supporting regions. Taking a Fourier transform breaks the population density into a sum of sinusoidal waves and enables us to quantify the signal strength at each wavelength. We do this for a 140 km line through Yorkshire and the Midlands, encompassing Leeds, Sheffield, Nottingham and Leicester. This is compared with a second slice taken through Bristol, Swindon, Oxford and Luton.

The results of both approaches can be seen in figure 2. In the North East region, we see a clear peak at a length of around 48 km and in Oxfordshire we see peaks at 43 km and 58 km. The Fourier transform shows similar dominant wavelengths in both regions of around 50 km.

As a brief comparison, appendix A shows similar analysis of length scales in the USA, which gives a longer characteristic length of around 200 km. Although these data are not entirely free from ambiguity, it shows that a characteristic length scale exists of around 45 km between cities in the UK and suggests that similar patterns but with different length scales may be found in different contexts.

## 4. Integro-differential equations for population and service dynamics

We model the spatio-temporal evolution of population density *p*(*x*,*t*) (number of residents per square kilometre) and service fraction, *s*(*x*,*t*) (fraction of land occupied by services), at location *x* and time *t*. Here, we assume services include all providers of employment, leisure, retail, etc. Defining services via land use, as opposed to an abstract term such as utility, has the advantage of tying in both with available data (table 1) and some cellular automata type models [22,24]. The model simulations will be in one or two spatial dimensions and the analysis is in one dimension. The difference between one and two dimensions will be the spatial kernels and the ease of computation.

**Table 1.** Table of parameters corresponding to equations (4.1) to (4.10) that define d*p*/d*t* and d*s*/d*t*. These parameters are based on UK data from publicly available data sources.

parameter	definition	default value	justification
*β*_*s*_	length scale for the kernel *w*_*s*_, characterizes how close services wish customers to be	5 km	mean trip distance to shopping in an urban conurbation is 4.7 km [33]
$\beta_{\;p_{1}}$	length scale used in kernel $w_{\;p_{1}}$ corresponding to how near population wishes to be to services	1 km	assume attraction of an area is given by local services; those within walking distance. Average walk to shops is 1.1 km [33]
$\beta_{\;p_{2}}$	length scale corresponding to how far population is willing to move to a more desirable location	10 km	in 2013–2014, the median household move was in the ‘5-10 miles’ group [34]
*λ*	population scale parameter for the carrying capacity function, equation (4.10)	20000 $\text{pe}\;{\text{km}}^{-2}$	in London wards 0 < *p* < 14 000 [35] and 0 < *s* < 0.4 (assuming *s* to be non-domestic land use) [36]. If *P* = *λ* then *s* will be expected to approach *σ*(*λ*) = 1 − (1/*e*) ≈ 0.63 which is significantly higher than the value for *s* in London. Since *σ*(*P*) is an increasing function, this means that *λ* must be larger than the maximum population density in London. Selecting *λ* = 20 000 seems a reasonable estimate for this upper end of population density and service provision
*μ*	steepness parameter for the carrying capacity function	3	taking the mean of London wards gives $\bar{\;p}=8400\;\text{pe}\;{\text{km}}^{-2}$ [35] and $\bar{s}=0.06$ (assuming *s* to be non-domestic land use) [36]. Using these values and solving the homogeneous steady-state equation (5.1) gives *μ* ≈ 3.2, assuming *λ* = 20 000
*D*	rate of change of population density in an area	$2\;{\text{yr}}^{-1}$	at the homogeneous state, the number of people who move in or out of a place is given by *D* · *p*_0_ · *A*_0_ = *D* · *p*_0_ · *σ*(*p*_0_) · (1 − *σ*(*p*_0_)). In London in 2018-19, flows in and out were approximately $290\;\text{pe}\;{\text{km}}^{-2}\;{\text{yr}}^{-1}$ [37] and population density, *p*_0_, was approximately $5700\;\text{pe}\;{\text{km}}^{-2}$. This gives *D* ≈ 2.2
*g*	speed of service followers per year	$2\;{\text{yr}}^{-1}$	ignoring *f*, d*s*/d*t* is at its maximum value when *σ* = 1 and *s* = 0.5, giving d*s*/d*t* = 0.25*g*. At this rate it will take 2/*g* years for *s* to reach *σ* = 1. *g* = 2 (1 year) seems a sensible timescale for this maximal rate
*f*	speed of service innovators per year	$0.05\;{\text{yr}}^{-1}$	we assume that change is mostly driven by logistic growth and therefore service innovation is slower than service followers. If *s* = 0, d*s*/d*t* = *f* · *σ*(*p*). At this rate it would take $\frac{1}{f}=20$ years to reach the steady state

We begin by defining a notion of *attractiveness* to the residential population, *A*(*x*,*t*), which captures the assumptions that a location is more attractive if there are services near to that place, but less attractive if the location is itself full of services (people may not want to reside in areas of dense service provision). *A* is then given as the product of a non-local average of service fraction and the local fraction of non-service space

4.1 A(x, t)=A(S(x, t), s(x, t))=S(x, t)(1−s(x, t)),

where

4.2 $$S(x,t)=\int w_{\;p_{1}}(x-y)s(y,t)dy=w_{\;p_{1}}*s(x,t).$$

Here, is a weight kernel, which captures the non-local contribution of service density. We assume a Gaussian kernel with length scale *β*_1_, so

4.3 $$w_{\;p_{1}}(x)=G(x,\beta_{1}),$$

where

$$\begin{array}{rl} & G(x,\;\beta )=\frac{1}{\beta \sqrt{2\pi }}\;e^{-(x^{2}/2\beta^{2})}\;\text{ (In 1D) }\\ \mathrm{and}\;\; & G(x,\;\beta )=\frac{1}{2\pi \beta^{2}}\;e^{-(|x{|}^{2}/2\beta^{2})}\;\text{ (In 2D) } \end{array}\}$$

Such a kernel means that points that are further away from *x* have less influence than points near *x*. It is used elsewhere in the urban modelling literature [16,38]. The rate of decay is given by *β* where larger *β* gives a more spread shape, synonymously with the standard deviation of a normal distribution. The parameter *β*_1_ therefore characterizes how near the residential population wishes to be to services.

We then assume that the rate of population movement from location *y* to *x* is proportional to the attractiveness of *x* multiplied by the density of potential movers at *y* and weighted according to the distance between the locations (short distance moves being more likely [34]). The rate of change in population at a location *x* will therefore be given by the rate of moving from all other locations *y* to *x* (moves into *x*), minus the rate of moving from *x* to all other locations *y* (moves out of *x*):

4.4 $$\frac{dp}{dt}(x,\;t)=D\int [A(x)p(y)-A(y)p(x)]w_{\;p_{2}}(x-y)\;dy,$$

where *D* measures the overall rate of moving and

4.5 $$w_{\;p_{2}}(x)=G(x,\beta_{2}),$$

gives a Gaussian dependence of the rate on the distance moved.

The dynamics of the service fraction, *s*(*x*,*t*), is assumed to be driven by a demand that is an increasing function of the residential population that can access services at *x*. Growth in the service fraction is therefore driven by *innovators* that start new businesses when demand exceeds supply, and expansion of existing businesses, *imitators*. However, if supply exceeds demand, then competition would force some out of business. We have

4.6 $$\frac{ds}{dt}(x,\;t)=(f+gs)(\sigma (P)-s).$$

The rates *f* and *g* represent the speed of service innovators and imitators respectively. The carrying capacity for services for a given population density is *σ*(*P*(*x*,*t*)). *P*(*x*,*t*) is a weighted integral of *p*(*x*,*t*), where the third and final kernel in the model, *w*_*s*_, encapsulates dependence of the carrying capacity on the population distribution. The kernel here captures the typical distance residents travel to places of work, retailers and other services. The equation for *P*(*x*,*t*) is

4.7 $$P(x,t)=w_{s}\;*\;p(x,t).$$

We assume the spatial weight kernel *w*_*s*_ is Gaussian; specifically

4.8 $$w_{s}(x)=G(x,\;\beta_{s}).$$

From the definition of *s*(*x*,*t*) being the fraction of land occupied by services, we require 0 ≤ *s* ≤ 1 and so 0 ≤ *σ* ≤ 1 also. It is natural to expect that the greater the population near *x*, the more services can be supported at *x* by this population, therefore *σ*(*P*) should be a non-decreasing function. We assume that carrying capacity for services will take the form

4.9 $$\sigma (P)=1-e^{-{\left(P/\lambda \right)}^{\mu }}.$$

This function has the features that *σ*(0) = 0, lim _*p*→∞_*σ*(*p*) = 1 and d*σ*/d*P* > 0. *σ* can be understood as the potential for service provision for a given population. The parameter *λ* represents the population scale and *μ* represents the shape of the function. If *μ* ≤ 1 this function is concave and if *μ* > 1 it is sigmoidal with maximum steepness increasing with *μ*. The sigmoidal form would model a situation in which, at low populations, the benefits of setting up a business barely exceed the fixed costs and service carrying capacity increases weakly. As the population increases, there may be a tipping point where the benefits gained increasingly outweigh the costs, leading to a marked steepening of the carrying capacity function, which then levels off as the population approaches saturation.

For simplicity, we assume initially that there will be no growth of the total population. Instead, we will analyse how the steady states of this model depend on the total population.

**Figure 3.**
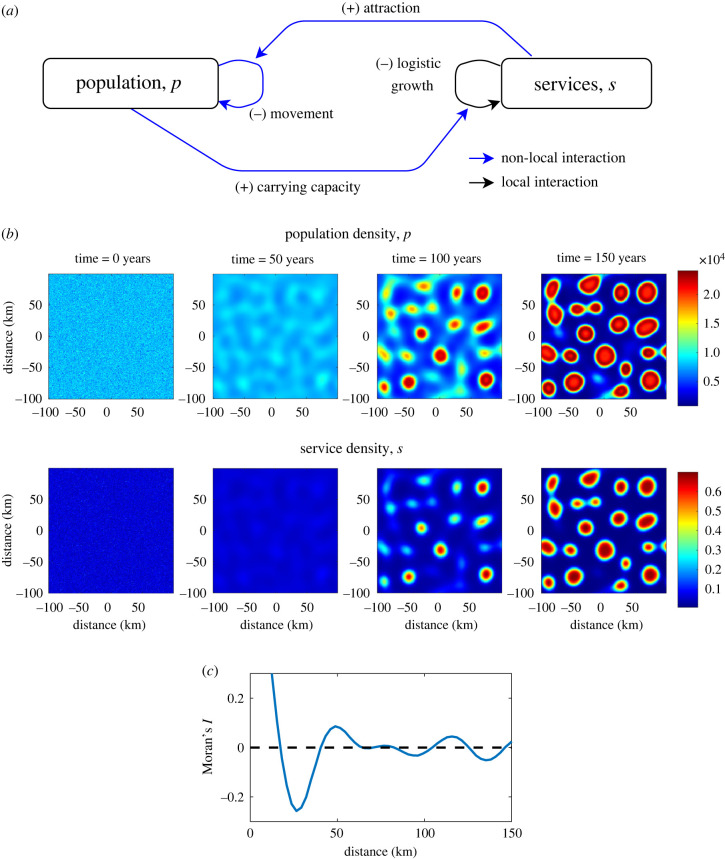
Emergence of cities from an initial random population distribution. (*a*) The key interactions in the model. *p* and *s* represent population and services at a point. Blue lines represent non-local dynamics, that is where a spatial convolution is used to characterize the influence of a distribution in the neighbourhood around a point. (*b*) We initialize a population of *p* = 8000 + 1000*r*, where *r* ∼ *N*(0, 1). After 100 years, we see the clear emergence of city structures, which are further strengthened at 150 years. (*c*) The length scale between these cities is around 53 km as can be seen in the autocorrelation plot of the final simulation. Parameters correspond to the default parameters in table 1.

Equations (4.4) and (4.6) give a description of the spatio-temporal interaction between population and services. A diagram explaining these interactions can be seen in figure 3*a*. Explanations and estimates for the default parameters corresponding to UK data are given in table 1. Numerical methods are explained in appendix B.

Simulations of the model with the kernels and parameters listed show that the system tends either to a spatially homogeneous state, where population and services are completely mixed, or spatial patterns emerge. These patterns take the form of areas of increased population and service density, as shown in figure 3*b*. An initial homogeneous population is seeded with small random perturbations. After 50 years, we start to see some areas growing more than others and, by 100 years, clear city structures have formed. The largest cities have a slight density crater in the city centre. Continuing the simulation to 150 years shows solidification of the city structure occurring as people move to the city. The length scale of the pattern is 53 km according to the spatial autocorrelation (figure 3c).

## 5. Spatial instability leads to patterned steady states

We wish to understand the conditions in which a homogeneous steady state or a spatial pattern emerge. For any homogeneous {*p*_0_, *s*_0_}, equation (4.4) will be zero and therefore the homogeneous steady state for *p* is $p_{0}=\bar{\;p}$, the average population density, which is dictated by the initial conditions. Equation (4.6) gives the homogeneous steady state for *s* as

5.1 $$s_{0}=\sigma (p_{0}).$$

To better understand which wavelengths we expect to emerge from an unstable homogeneous state, we consider the perturbation from the steady state

5.2 $$p=p_{0}+\tilde{\;p}(x,t),s=s_{0}+\tilde{s}(x,t).$$

In particular, we look for Turing-like instabilities; that is by looking at sinusoidal perturbations of frequency *k*, given by

5.3 $$\{\tilde{\;p}(x,t),\tilde{s}(x,t)\}=\{\overset{ˇ}{\;p}(t)\;e^{ikx},\overset{ˇ}{s}(t)\;e^{ikx}\}.$$

Linearizing, we obtain a problem of the form

5.4 $$\left(\begin{array}{c} \dot{\overset{ˇ}{\;p}}\\ \dot{\overset{ˇ}{s}} \end{array}\right)=J(p_{0},s_{0},k)\left(\begin{array}{c} \overset{ˇ}{\;p}\\ \overset{ˇ}{s} \end{array}\right)$$

so that we can analyse the stability matrix *J*. Calculation of *J* can be found in appendix C.1, giving

5.5 $$\begin{array}{c} J(\;p_{0},\;s_{0},\;k)\;\\ \begin{array}{l} \;\;=\left[\begin{array}{cc} -Ds_{0}(1-s_{0})(1-{\hat{w}}_{\;p_{2}}(k))\; & Dp_{0}(1-{\hat{w}}_{\;p_{2}}(k))\;\cdot \;\\ \; & \left((1-s_{0}){\hat{w}}_{\;p_{1}}(k)-s_{0}\right)\;\\ \left(\;f+gs_{0})\sigma^{\prime }(\;p_{0}){\hat{w}}_{s}(k\right)\; & -(\;f+gs_{0}) \end{array}\right]. \end{array} \end{array}$$

**Figure 4.**
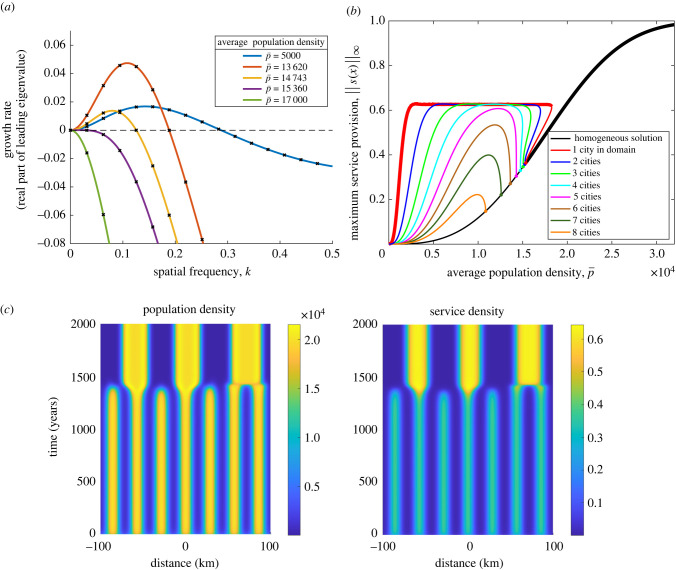
(*a*) Dispersion relations at five different values of the average population, $\bar{\;p}$. The crosses correspond to the different wavenumbers. This diagram shows which spatial frequencies are unstable; the larger the real part of the eigenvalue, the more destabilizing that frequency. As population density $\bar{p}$ increases, the length scale of the dominant unstable wavelength increases from around 40 km (k=0.15) to around 60 km (k=0.1). At $\bar{\;p}=15\;360,$ the dispersion relation is tangential to the horizontal at *k* = 0. This is the bifurcation point where no modes are unstable. At $\bar{\;p}>15\;360$ any perturbation decays. (*b*) Bifurcation diagram showing where patterned steady states exist as average population density varies. Thick lines correspond to stable solutions and thin lines correspond to unstable solutions. The stable branch is the single city solution, but other branches display metastability. (*c*) Time simulation showing how a seven bump metastable steady state suddenly transitions into a three bump state after a very long time period. The three bump solution is itself metastable but persists for any reasonable timescales. Initial conditions were *p*(*x*,0) = 10000 + 5000 cos (7*πx*/100) with *s*(*x*,0) = *σ*(*p*(*x*,0)).

For each frequency, *k*, perturbations are unstable if at least one eigenvalue of *J*(*k*) has positive real part. We plot the real part of the leading eigenvalue against spatial frequency in dispersion relations to see the modes with a positive growth rate. We plot these dispersion relations as we vary the average population in the model in figure 4*a*.

This shows that for small populations, modes of lower frequency grow, but higher frequency, shorter wavelength modes dissipate. As population density increases, the fastest growing mode moves from five peaks in the domain (40 km wavelength) to 2–3 peaks (60–100 km). For larger population densities, the dispersion relation predicts that all perturbations will dissipate.

We can further analyse this Jacobian to calculate conditions for any wavelengths to be unstable. We show in appendix C.1.1 that there are unstable frequencies if and only if

5.6 $$\frac{s_{0}(1-s_{0})}{p_{0}\sigma^{\prime }(p_{0})}+s_{0}<(1-s_{0}),$$

and that, if this holds, then instability occurs in a window (0, *k*_*c*_) for some critical frequency *k*_*c*_.

We can solve for equality in (5.6) using (5.1) to find the bifurcation point at which spatial instability arises, {*p*0, *s*0}. For the carrying capacity function given by equation (4.9), the bifurcation point in *s* occurs at the solution of *s*_0_ = *μ* (2*s*_0_ − 1) ln(1 − *s*_0_). The bifurcation point depends only on *μ* and the equation has a solution for *μ* ≥ 1. The parameter *μ* is the steepness of the change in carrying capacity for services for a change in population. It is concave with respect to population density if *μ* < 1 and sigmoidal otherwise. Using the value of *μ* = 3 in figure 4, we calculate *s*_0_ = 0.37, *p*_0_ = 15 360 for the bifurcation point.

For a more general *σ*(*p*), if equation (5.6) holds, we must have *p*_0_*σ*′(*p*_0_) > *s*_0_ = *σ*(*p*_0_). Although technical exceptions can be found, this suggests that *σ* should be convex on (0, *p*_0_) for instabilities to the homogeneous steady state to arise. This model analysis predicts that when service potential is highly reactive to a change in population (convex *σ*(*p*)) then we expect pattern generating instabilities to arise. Conversely, if a change in population does not drive a sufficient reaction in service potential then we expect such a perturbation to die away.

Moreover, if equation (5.6) holds and there are instabilities of the homogeneous steady state, then . Regardless of the carrying capacity function, instability can only occur where we have sufficiently small population densities and services. If the population is too high, the model predicts that there would be urban sprawl rather than further agglomeration.

We can also see that for a solution to be unstable to a certain perturbation, that perturbation must have a sufficiently long wavelength. In an urban context, this means that we do not expect to see lots of very small but high-density cities next to each other. In this case, we expect agglomeration would occur. However, if cities are far enough apart, we can expect them to remain distinct.

Having analysed the homogeneous steady state, we can now look at the patterned steady states and determine their stability. These can be calculated using a process of numerical continuation [39]. Beginning with a steady state produced by simulation, we track how this state changes as we vary the average population size, $\bar{\;p}$. This allows us to see the regions in which a patterned state can exist and to explore the bifurcation points. This can be seen in figure 4*b*. The patterned states emerge at low average populations until a series of bifurcation points where each becomes stable. The last bifurcation to the homogeneous state is at confirming what we saw with the dispersion relation. Between $\bar{\;p}=15\;360$ and $\bar{\;p}=18\;000$, both the homogeneous steady state and the patterned state are stable and for $\bar{\;p}>18\;000$ only the homogeneous state is stable.

Figure 4*b* suggests that the only stable patterned state is the single cluster pattern. This is due to the presence of metastable steady states, where the instability can only be seen by simulating the system for an extreme length of time, as in figure 4*c*. All the multi-peak branches display this metastability. However, the timescales at which this agglomeration occurs might be of the order of greater than a millennium or even more and these steady states are therefore only very weakly unstable.

We have shown how distinct cities can form where the average population density is sufficiently low and there is sufficient reaction by services to a change in population. Unstable perturbations to the homogeneous steady state must be of sufficiently long length scale showing how we expect agglomeration to occur over longer rather than shorter distances. The dominant unstable wavelength of such perturbations can be seen in the dispersion relations given by the linear stability analysis of the steady state. We also saw the presence of bifurcation points and metastable steady states in the system which shows how sudden agglomeration may occur, particularly as population increases.

## 6. Secondary patterning when services compete for space and residents avoid high service density

London in figure 1 shows an example of multiple patterning. Firstly, aggregation brings people and services together to form the city itself and there is a growth in population density towards the city centre. However, there is secondary patchy patterning on top of this framework. This may be driven by people and services occupying distinct areas (one is high when the other is low)—seen in particular at the centre of the city.

Within our model framework, we make two further assumptions. We assume that people’s desire is to locate ‘near but not too near’ to the services that support their needs; and that there is competition for space between people and services within cities. The first assumption about people’s location choice can be built into kernel $w_{\;p_{1}}$ by using a Gaussian kernel that has been shifted off centre by distance *a*_*p*_ in each direction and then summed

6.1 $$w_{\;p_{1}}(x)=\frac{1}{2}(G(x+a_{p},\;\beta_{\;p_{1}})+G(x-a_{p},\;\beta_{\;p_{1}})).\;$$

*G* is the Gaussian previously defined in equation (4.4). This assumes that there is an ideal distance *a*_*p*_ which people wish to be from service locations. Moreover, competition is introduced into equation (4.6) as services compete for space with residents.

6.2 $$\begin{array}{rl} \frac{ds}{dt}(x,\;t) & =(H(\sigma (P)-(s+\alpha_{1}p))\;f+gs)\cdot (\sigma (P)-(s+\alpha_{1}p)). \end{array}$$

With competition now included, the space requirements of people can overcome the potential for service growth so we assume that if there is no potential for service growth then there are no innovators, *f*. This is why we have a factor of *H*(*σ*(*P*) − (*s* + *α*_1_*p*)), where *H* is the Heaviside step function. Importantly, this ensures that *s* = 0 is a lower bound for services. The default values for the new parameters *a*_*p*_ and *α*_1_ can now be found in table 2.

**Table 2.** Supplement to table 1. Additional parameters for equations (6.1) and (6.2).

parameter	definition	default value	justification
*a*_*p*_	secondary length scale used in kernel $w_{\;p_{1}}$ corresponding to the population's preferred distance to services	1.5 km	given $\beta_{\;p_{1}}=1\;\text{km}$, *a*_*p*_ = 1.5 creates distinct, off centre, peaks without separating the two Gaussians completely
*α*_1_	competition parameter. How much space does each person take away from services	1.5 × 10^−5^ ${\text{km}}^{2}\;{\text{pe}}^{-1}$	when population dominates, *α*_1_ *p* = 1. In London wards, the maximum population density is $28\;863\;\text{pe}\;{\text{km}}^{-2}$ which would give $\alpha_{1}=3.53\times 10^{-5}\;{\text{km}}^{2}\;{\text{pe}}^{-1}$. We assume less competition than this [35]

**Figure 5.**
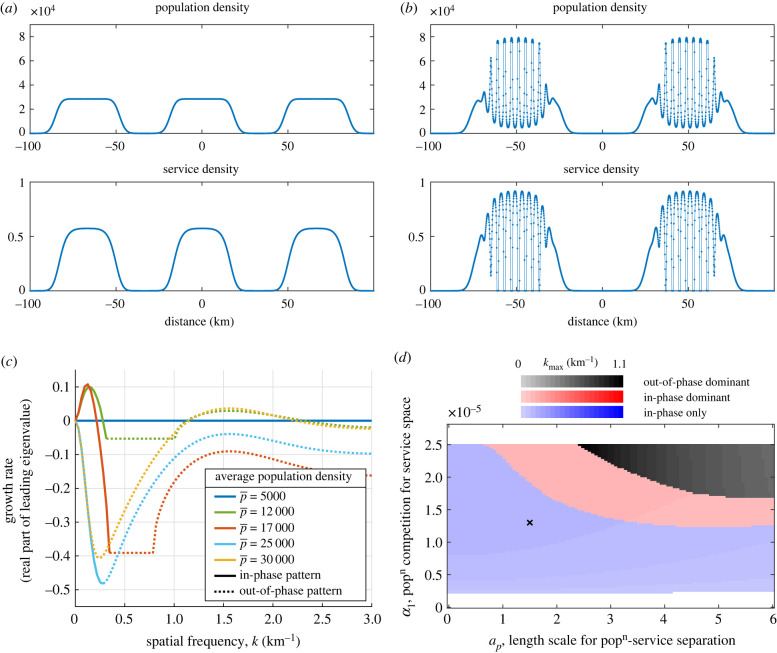
Out-of-phase patterning for the model with competition. (*a*,*b*) Example simulations showing how in-phase and out-of-phase patterning are possible. Both have the same parameters and mean population $\bar{\;p}=16\;000$, with *α*_1_ = 1.3 × 10^−5^. Initial conditions can be seen in figure 11. (*c*) Dispersion relations for different values of the average population, $\bar{\;p}$. At low populations, there is not sufficient service potential to overcome competition for space so no instability can persist. At higher populations, both long wavelength in phase patterns and short wavelength out-of-phase patterns are predicted by the analysis. Finally, at higher population densities, only out-of-phase patterning persists. (*d*) Spatial instabilities, their phase and spatial frequency predicted by the linear analysis in the *a*_*p*_, *α*_1_ parameter-space. We use for which the spatially homogeneous steady state is stable when *a*_*p*_, = 0 = *α*_1_. For weak competition (*α*_1_ > 2.5 × 10^−6^) an in-phase instability of low frequency (long wavelength) occurs. For sufficiently strong competition and desire for spatial separation, out-of-phase instability with higher spatial frequency is dominant. For *α*_1_ > 2.5 × 10^−5^, there is so much competition for space that services do not have the population nearby to overcome this competition and they die out completely, leaving a stable solution again. The black ‘x’ marks the point in parameter space used for the simulations in (*a*,*b*) showing how secondary patterning persists even into regions not predicted by the linear stability analysis.

Including competition can give both in-phase and out-of-phase patterning at different spatial scales (figure 5*a*,*b*). In-phase patterns are the co-location of high-densities of people and services, such as seen on a large scale in cities. Out-of-phase patterns are where people and services occupy distinct and complementary areas; here they typically have shorter length scales and occur as secondary structures within cities.

In this example, a three bump solution with no secondary pattern (figure 5*a*) persists at the same parameter values as a two bump solution with shorter wavelength secondary patterning within each bump (figure 5*b*). This secondary pattern has a wavelength of around 5 km.

In order to understand the emergent length scales, we again use linear stability analysis around the homogeneous steady state, {*p*_0_, *s*_0_}. The steady state for *p* is given by $p_{0}=\bar{\;p}$, the average population density which is dictated by initial conditions. For smaller population densities ($\bar{\;p}<11\;500$), we have that *σ*(*p*_0_) < *α*_1_*p*_0_ and so the spatially homogeneous steady state is $p_{0}=\bar{\;p}$, *s*_0_ = 0. There will not be sufficient demand for services to overcome the competition for space and so no services can be supported. Patterned states can exist with these average population densities but they are not emergent from the homogeneous steady state. Instead, they would have to emerge from different initial conditions.

For $\bar{\;p}>11\;500$, we have that *σ*(*p*_0_) > *α*_1_*p*_0_ and the steady state is

6.3 $$p_{0}=\bar{\;p},\;s_{0}=\sigma (p_{0})-\alpha_{1}p_{0}.$$

The Jacobian is given by

6.4 $$\begin{array}{c} J(\;p_{0},\;s_{0},\;k)\;\\ \;\;=\left[\begin{array}{cc} -Ds_{0}(1-s_{0})(1-{\hat{w}}_{\;p_{2}}(k))\; & Dp_{0}(1-{\hat{w}}_{\;p_{2}}(k))\;\cdot \;\\ \; & \left((1-s_{0}){\hat{w}}_{\;p_{1}}(k)-s_{0}\right)\;\\ \left(\;f+gs_{0})(\sigma^{\prime }(\;p_{0}){\hat{w}}_{s}(k)-\alpha_{1}\right)\; & -(\;f+gs_{0}) \end{array}\right]. \end{array}$$

The Jacobian not only enables us to see whether an instability emerges but we can also see the phase of this perturbation; whether we expect *p* and *s* to grow in the same places or to separate. We do this by looking at the eigenvector corresponding to the unstable eigenvalue. Figure 5*c* shows the change in the dispersion relation as we increase the initial population. At $\bar{\;p}=5000,$ the homogeneous state is given as *s*_0_ = 0. At $\bar{\;p}=12\;000$, both in- and out-of-phase patterning are predicted. As we increase the population density to no pattern is unstable. Lastly, as the population density further increases, the competition forces out of phase patterning again. Moreover, we see that in phase patterning occurs at similar frequencies to before, with a wavelength  of around 50 km. Out-of-phase patterning occurs at a shorter wavelength of around 5 km, which is a frequency of 1.25. In order for out-of-phase patterns to be predicted by the linear analysis, we must have *α*_1_ > 0 and *a*_*p*_ > 0 (appendix C.2). These are necessary but not sufficient conditions.

Figure 5*d* maps an example of where in the *α*_1_, *a*_*p*_ parameter regime in- and out-of-phase patterning is predicted. The homogeneous steady state is given by *s*_0_ = *σ*(*p*_0_) − *α*_1_*p*_0_. Therefore, as we increase *α*_1_ so the steady state for *s*_0_ will decrease. If the original homogeneous steady state is stable, as in figure 5, introducing some competition may induce in-phase patterning. Increasing *a*_*p*_, the ideal length people wish to be from services, can induce out-of-phase patterning, as long as there is sufficient competition to drive it. For very large *α*_1_, competition for space means that services do not have the population nearby to overcome this competition and they die out completely, leaving a stable steady state again.

The out-of-phase patterning seen in figure 5*b* is not predicted by the linear stability analysis of the homogeneous steady state for this average population yet it can persist across the parameter space. In this particular case, such patterning is secondary, forming after an initial city has grown and developed.

In summary, this model produces both in- and out-of-phase patterns of different spatial lengths; the new out-of-phase patterning is of shorter wavelength. Within a city context, the desire for co-location agglomerates people into cities and competition for space creates divisions in land use.

## 7. Including population growth and competition shows cities emerging before secondary patterning appears

Lastly, we include population growth in the model via logistic growth up to some carrying capacity with competition for space from services. This changes equation (4.4) to be

7.1 $$\frac{dp}{dt}(x,\;t)=D\int [A(x)p(y)-A(y)p(x)]w_{p_{2}}(x-y)\;dy+rp\left(1-\frac{\;p+\alpha_{2}s}{c}\right).$$

**Figure 6.**
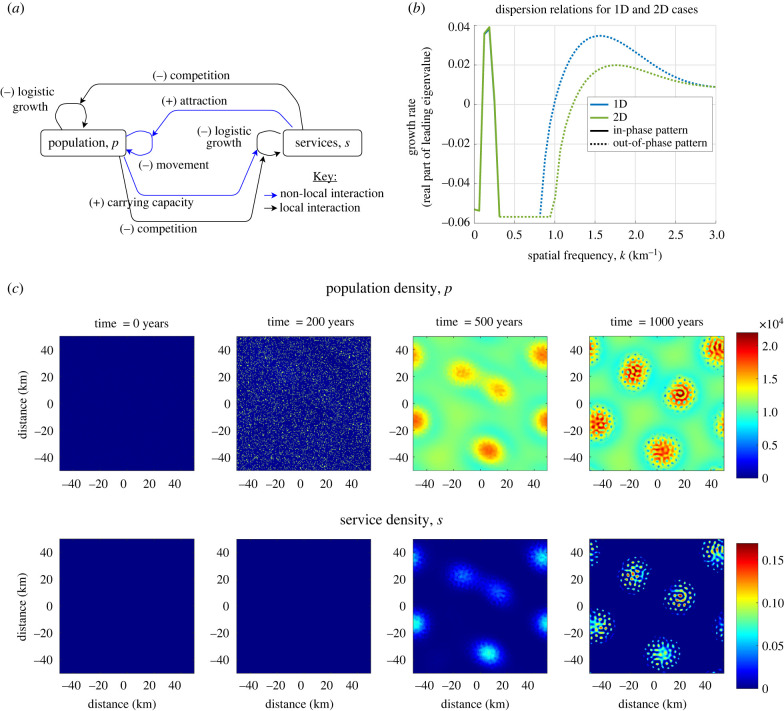
(*a*) Schematic diagram of the full model from §7 explaining the key interactions. This encompasses equations (6.2) and (7.1). (*b*) Dispersion relations corresponding to the one-dimensional and two-dimensional cases for the default parameter set (tables 1, 2 & 3), also corresponding to the simulation in (*c*). This shows that both in- and out-of-phase patterning can still be predicted when both the population and services grow and compete for space. (*c*) Simulation of the growth and secondary pattern formation of cities. Initial conditions are *p*_0_(*x*, *y*) = 200 on 10% of the domain. We see that, firstly, population grows everywhere and as it does, distinct cities appear. Then secondary structure emerges within the cities as population separates from services.

A schematic of the full model can be seen in figure 6*a* and the new parameter values in table 3 with explanations in appendix E.

**Table 3.** Supplement to tables 1 and 2 showing the default values for the new parameters in equation (7.1)

parameter	definition	default value	justification
*r*	intrinsic population growth rate	$0.05\;{\text{yr}}^{-1}$	see appendix E
*c*	carrying capacity for population density	12 000 $\text{pe}\;{\text{km}}^{-2}$	*c* will define *p*, therefore we consider a similar range
*α*_2_	competition of services to population density	100 000 $\text{pe}\;{\text{km}}^{-2}$	at steady state, *p* + *α*_2_*s* = *c*. If $p=10\;000\;\text{pe}\;{\text{km}}^{-2}$, and *s* = 0.05, then $\alpha_{2}=100\;000\;\text{pe}\;{\text{km}}^{-2}$

Similarly to the previous case, in- and out-of-phase patterning are possible, depending upon the value for the carrying capacity *c*. This can be seen in dispersion relations as we vary the carrying capacity in figure 10 and in the example in figure 6. The homogeneous steady state from d*p*/d*t* is now dependent upon the carrying capacity *c*. The steady state is now given by the solution to

7.2 $$p_{0}+\alpha_{2}s_{0}=c,\;s_{0}=\sigma (p_{0})-\alpha_{1}p_{0}.$$

We note that it is possible to keep the previous steady state {*p*_0_, *s*_0_} unchanged by choosing *c* as

7.3 $$c=p_{0}+\alpha_{2}s_{0}.$$

The Jacobian used to produce these dispersion relations is as follows:

7.4 $$\begin{array}{c} J(\;p_{0},\;s_{0},\;k)\;\\ \;=\left[\begin{array}{cc} \; & Dp_{0}(1-{\hat{w}}_{\;p_{2}}(k))\;\cdot \;\\ -Ds_{0}(1-s_{0})(1-{\hat{w}}_{\;p_{2}}(k))-\frac{rp_{0}}{c}\; & ((1-s_{0}){\hat{w}}_{\;p_{1}}(k)-s_{0})\;\\ \; & -\frac{r\alpha_{2}p_{0}}{c}\;\\ \left(\;f+gs_{0})(\sigma^{\prime }(\;p_{0}){\hat{w}}_{s}(k)-\alpha_{1}\right)\; & -(\;f+gs_{0}) \end{array}\right]. \end{array}$$

The effect of logistic growth is generally stabilizing for long wavelength perturbations. In particular, homogeneous perturbations would return to the steady state. However, the competition parameter *α*_2_ will tend to be destabilizing for higher spatial frequencies, assuming that population and services both compete sufficiently for space (appendix C.3).

Figure 6*c* shows an example simulation of the full model in two dimensions, demonstrating growth of cities and secondary, out-of-phase, patterning that gives separation of population and services. Population initially grows but is not sufficient to drive urbanization. Then, after a number of years, cities form as there is sufficient population to drive the demand for services and colocation. Continuing time forward further shows that, within these cities there are distinct areas of service provision, surrounded by population. This model more realistically captures the long-term growth dynamics of population into urban areas.

## 8. Discussion

### 8.1. Conclusion

The emergence of spatial structure in human populations has received relatively little attention when compared with the quantification of urban patterns. Much focus has been on measuring structure rather than understanding or predicting where those structures come from. Inspired by typical length scales that are apparent in population density data between cities, here we have shown that a simple set of plausible local and spatial interactions can explain the emergence of cities via reinforced aggregation. While conceptually simple in comparison with computer modelling techniques such as cellular automata and agent-based models, these models benefit from deeper explanatory power; offering the potential not just to describe what we currently see in cities but also to explain how such dynamics emerge.

The hierarchy of integro-differential equation models developed here focuses on spatial kernels to capture the distribution of non-local dependencies. This model shows that the preference for population location in proximity to services can either lead to a completely mixed homogeneous state or drive the emergence of urban centres, seen in a spatial pattern. Numerical continuation and linear stability analysis of the steady states of this model shows how different length scales emerge, depending upon the initial conditions and parameters (figure 4). In phase spatial instabilities are shown to be destabilizing only if the perturbation is of a sufficiently long spatial scale and only if a change in population density produces a sufficient change in service density. One observation from this model is that many steady states are metastable; over long time periods, we would see transitions at the merging of city centres as cities agglomerate.

Within cities, we also see patterning emerge around local services as long scale co-location and short scale separation of population and service provision occurs. In the model, this is driven by competition for space and desire by people to be near, but not too near, to the services they need to support them (figure 5).

Length scales within the model are typical of those seen in the data for the UK. Differences in parameters such as house moves and preferred travel distances may explain different length scales between the UK and the USA. In the USA, people might tend to move greater distances, both to move house and to travel to their desired services, which gives rise to sprawling metropolises. Conversely, the rapidly urbanizing cities of China and Brazil may be driven by people moving long distances to be as close as possible to services, generating high-density distinct megacities. Our modelling approach would give valuable insight into the different city formations around the world.

### 8.2. Limitations and future developments

Our parsimonious approach has several limitations. Firstly, by using an aggregated differential equation methodology, we have gained mathematical tractability but lost the effects of population heterogeneity. As explained in the literature review, we believe that this approach provides a useful counterpoint to the increasingly popular bottom up agent-based methodologies.

**Figure 7.**
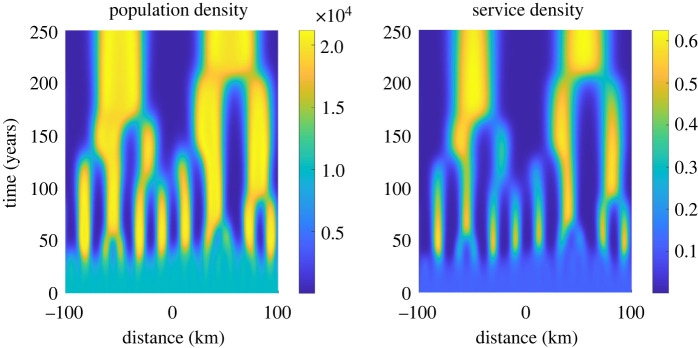
Increasing typical travel distances over time leads to exaggerated agglomeration. We vary $\beta_{\;p_{1}}$, $\beta_{\;p_{2}}$ and *β*_*s*_ as in (F1). The mean population density is $\bar{\;p}=10\;000\;\text{pe}\;{\text{km}}^{-2}$. Initially (*t* ≈ 30), the pattern that emerges from random perturbations to the spatially homogeneous steady state has a wavelength of approximately 16 km. As time continues, the pattern coarsens and larger agglomerations form with a wavelength of about 100 km. Further details of the parameter variation can be found in appendix F.

**Figure 10.**
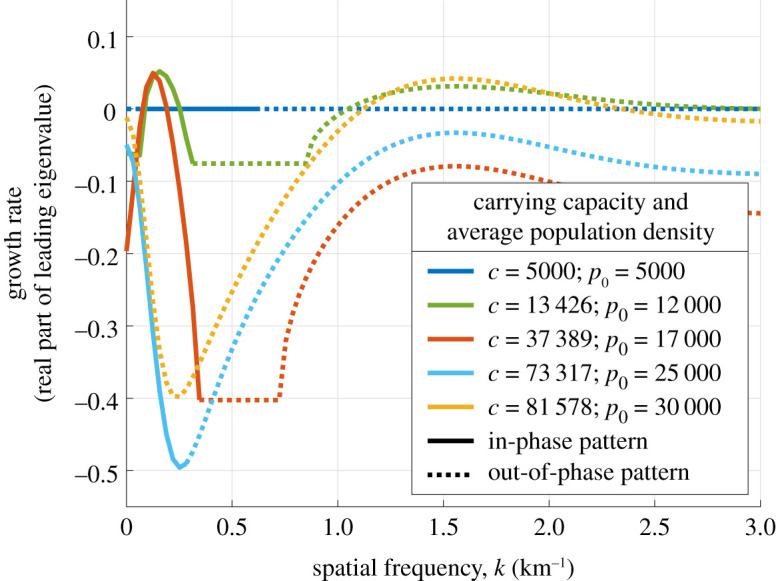
Dispersion relations for the full model including logistic growth and competition for space. This figure is very similar in its trend to figure 5*c*. The most notable difference is that there is no longer a zero eigenvalue at *k* = 0 corresponding to conservation of mass.

Secondly, we have considered parameters that are static over time. Technological advances and societal changes will doubtless affect the functions and kernels that capture behaviour. This simplification enables us to provide mathematical rigour to our conclusions that would not be possible with further complicating assumptions. Such work is beyond the scope of this paper, but we motivate the discussion with one example of dynamically changing length scale parameters in figure 7 to highlight the potential of our modelling approach.

Thirdly, we have assumed that people (and services) are motivated solely by coexistence preferences. In reality, people’s desires will also be affected by multiple competing interests such as employment opportunities, housing stock and house prices, transport and more—all of which require attention. This would be further confounded by the disaggregation of people according to factors such as income or ethnicity, or disaggregation of service types into retail, industry, etc. It is not easy to determine the relative importance of such influences or disentangle the effects of each. Our focus on colocation preferences of people and services has enabled us to elicit understanding regarding the implications of those preferences for patterning and urban length scales.

Despite these limitations, our approach is intuitive and mathematically rigorous. By developing an aggregated form of model, we have sidestepped the myriad of uncertainties involved in bottom-up modelling and minimized the variability. We hope that this exploration can be a springboard for future developments to accommodate these complicating factors which could deepen our understanding of the spatial development of populations.

## Figures and Tables

**Figure 8.**
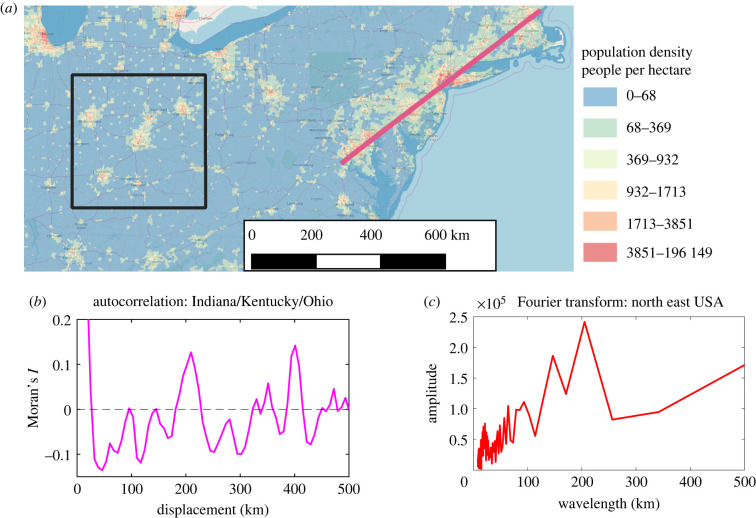
(*a*) Map of the population density of north east USA showing the areas analysed. Background mapping © Open street map contributors. (*b*) Correlogram for Indiana/Kentucky/Ohio region showing length scale peaks at 200 km and 400 km. (*c*) Fourier transform of the line from Washington to Boston, giving a corresponding wavelength of about 200 km.

**Figure 9.**
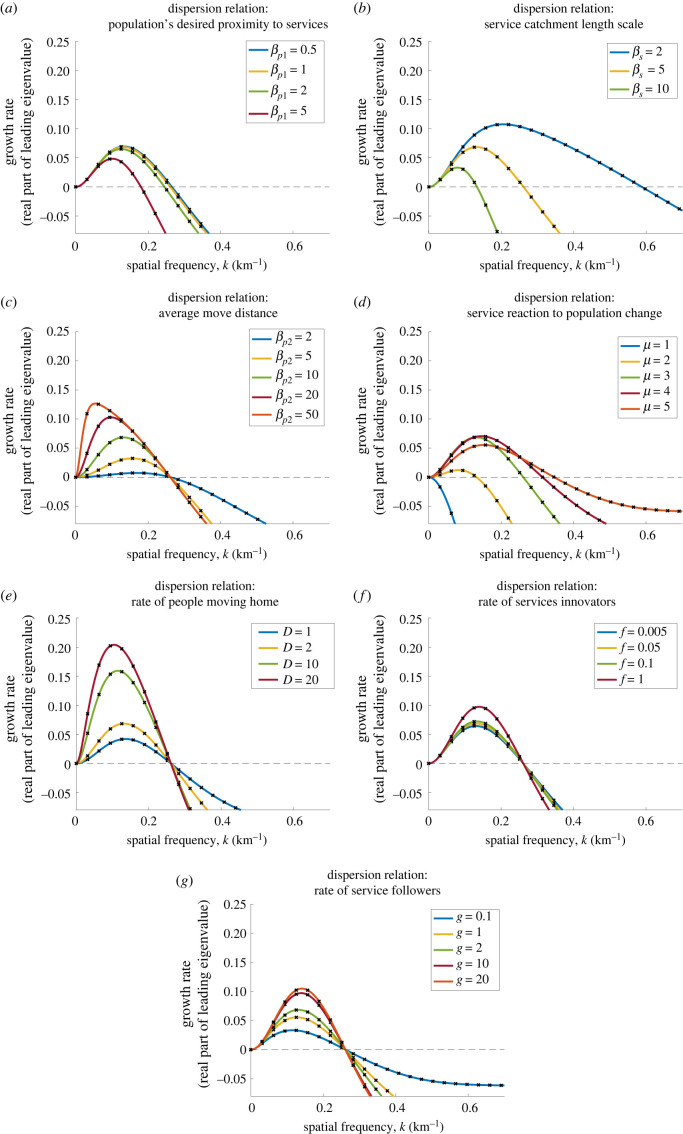
Dispersion relations for the growth rate of spatial perturbations to a spatially homogeneous steady state in the conservative model for population and services dynamics given in §4, for the indicated values of the parameters $\beta_{\;p_{1}},\beta_{s},\beta_{\;p_{2}},\mu ,D,f$ and *g*. For all length scales *β*, as they increase the length of pattern increases. Increasing $\beta_{\;p_{1}}$ and *β*_*s*_ slows the rate of patterning whereas $\beta_{\;p_{2}}$ increases the speed of patterning. As shown in §5, small values of *μ* show stability to all frequencies. As *μ* increases, the instability emerges from the origin with long wavelengths (small *k*) and shifts to shorter wavelengths (larger *k*). The rates *f*, *D* and *g* will, as expected, increase the growth rate of perturbations, but do not make any noteworthy change to the wavelength of the fastest growing mode or to the window of unstable frequencies.

**Figure 11.**
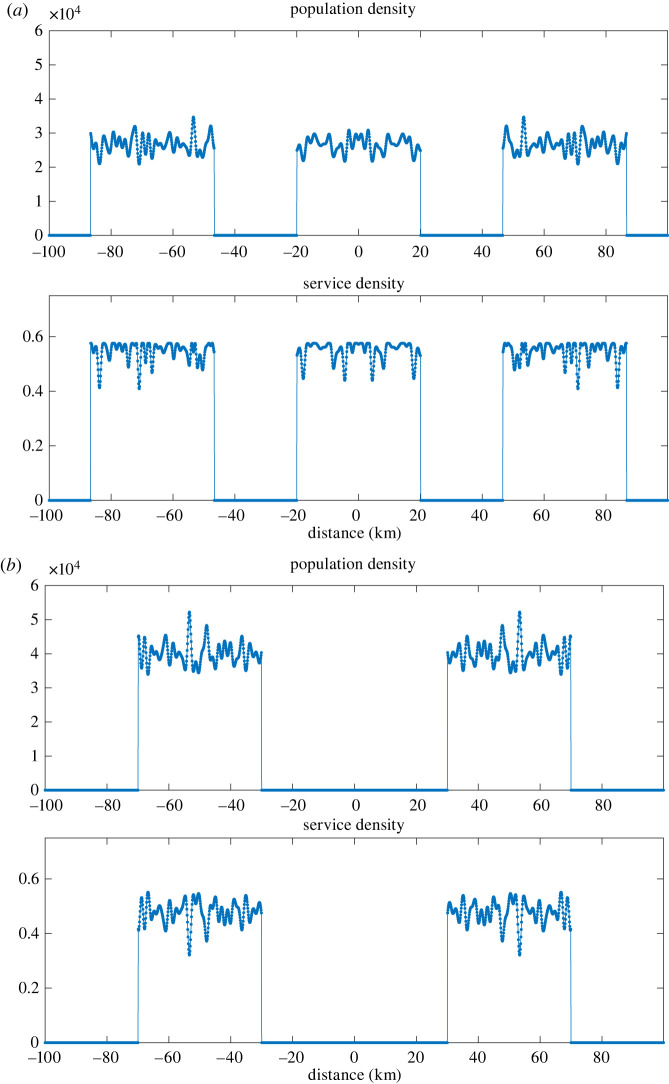
Initial conditions that generated figure 5*a*,*b*, respectively. These two initial conditions both have the same total population. However, including competition in the model, one simulation gives in phase patterns only whereas the other gives secondary out-of-phase patterning.

**Figure 12.**
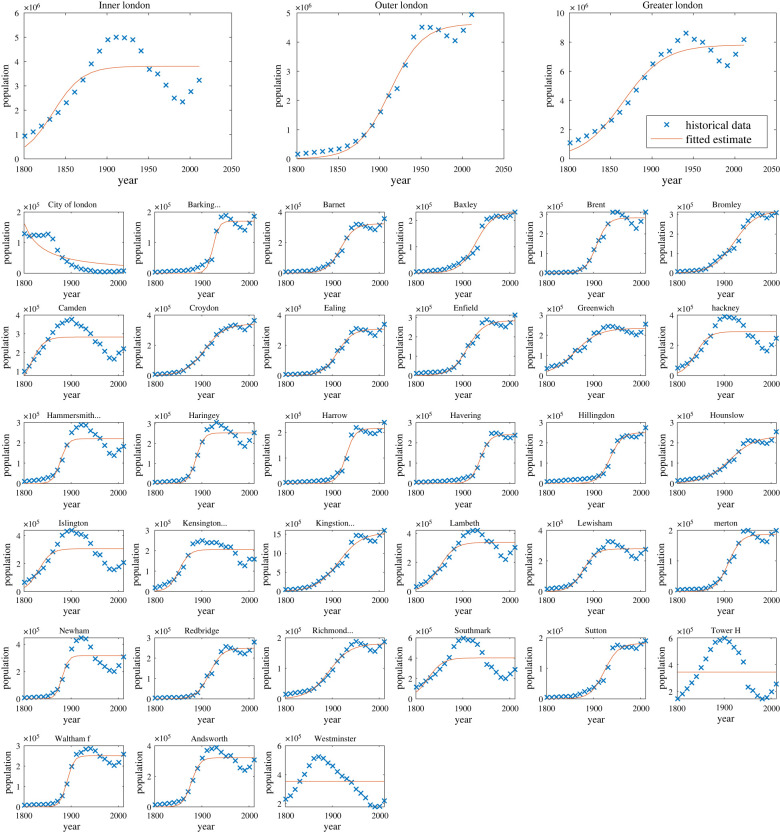
Graphs showing population data for London with the logistic growth model fitted.

## Data Availability

All data are from publicly available and cited sources. All code is publicly available at: https://doi.org/10.5281/zenodo.5034211.

## Appendix A. Length scales in the USA

Figure 8 shows the results from length scale analysis of population density data from the north east USA, from the 2010 Census at census tract level [40] similarly to §3. Autocorrelation on a region of Indiana, Ohio and Kentucky demonstrates a spatial length scale of 200 km. We also take the Fourier transform of a slice through key cities on the East coast: Washington, Philadelphia, New York and Boston, which gives a similar 200 km length scale. The USA seems to show a longer characteristic length scale than the UK.

## Appendix B. Numerical methods

For timestepping and numerical continuation, we use a code structure from Avitabile [39]. We use a pseudospectral collocation method to compute convolutional integral operators. Hence, we expect spectral accuracy for the spatial discretization (albeit we have not used de-aliasing). This requires the use of periodic boundary conditions; implying that the region considered is similar to its neighbouring regions.

To converge on and continue steady-states, we perform Newton-GMRES iterations with tolerance 0.001. For the time-stepper, we use the same spatial grid and discretization method as the steady-state calculations, and we employ Matlab's in-built ode113 routine, with default tolerances.

## Appendix C. Mathematical analysis of the model

**C.1. Linearization of the model without competition or population growth**

Here, we show the linearization of $\dot{\;p},\dot{s}$ from equations (4.4) and (4.6) for use in the Numerical continuation algorithm and then obtaining the Jacobian used in the linear stability analysis. We define

C 1 $$F(p,s)=\left(\begin{array}{c} \dot{\;p}(x,t)\\ \dot{s}(x,t) \end{array}\right).$$

Then we calculate $F(p_{0}+\tilde{\;p},s_{0}+\tilde{s})-F(p_{0},s_{0})$ where {*p*_0_, *s*_0_} are the current state and $\left\{\tilde{\;p},\tilde{s}\right\}$ are the perturbations from this state of order *ε*. Using a Taylor expansion for *σ*, we get

C 2 $$\begin{array}{rl} & \frac{F(p_{0}+\tilde{\;p},s_{0}+\tilde{s})-F(p_{0},s_{0})}{\epsilon }\\ & =\left(\begin{array}{c} D[A_{0}\cdot (w_{\;p_{2}}\;*\;\tilde{\;p})+\tilde{A}\cdot (w_{\;p_{2}}\;*\;p_{0})-\tilde{\;p}\cdot (w_{\;p_{2}}\;*\;A_{0})-p_{0}\cdot (w_{\;p_{2}}\;*\;\tilde{A})])\\ \left(f+g\cdot s_{0})\cdot (\tilde{\;p}\cdot \sigma^{\prime }(p_{0})-\tilde{s})+g\cdot \tilde{s}(\sigma (p_{0})-s_{0}\right) \end{array}\right), \end{array}$$

where

C 3 $$\begin{array}{rl} A & =A_{0}+\tilde{A}\\ & =(w_{\;p_{1}}*\;s_{0})\cdot (1-s_{0})+((w_{\;p_{1}}*\;\tilde{s})\cdot (1-s_{0})-(w_{\;p1}*\;s_{0})\cdot \tilde{s}). \end{array}$$

In order to look at different spatial frequencies, we consider perturbations that are sinusoidal by considering

C 4 $$\{\tilde{\;p}(x,\;t),\tilde{s}(x,\;t)\}=\{\overset{ˇ}{\;p}(t)\;e^{ikx},\overset{ˇ}{s}(t)\;e^{ikx}\}.$$

We will use the fact that the Fourier transform of a Gaussian kernel, as defined in (4.9), is $\hat{w}(k)=e^{-2(\pi^{2}\beta^{2})k^{2}}$. This is also Gaussian and $\hat{w}(0)=1$ for any *β*. It has a maximum at *k* = 0 and $\forall k,0\leq \hat{w}(k)\leq 1$. Moreover, we define the kernel $w_{\;p_{12}}\;:=w_{\;p_{1}}\;*\;w_{\;p_{2}}$. In the case that $w_{\;p_{1}}$ and $w_{\;p_{2}}$ are Gaussian, the convolution theorem gives that: $F(w_{\;p_{12}})=F(w_{\;p_{1}})F(w_{\;p_{2}})=e^{-2(\pi^{2}\beta_{\;p_{1}}^{2})k^{2}}\;e^{-2(\pi^{2}\beta_{\;p_{2}}^{2})k^{2}}=$ $e^{-2\pi^{2}(\beta_{\;p_{1}}^{2}+\beta_{\;p_{2}}^{2})k^{2}}$. Thus $w_{\;p_{12}}$ is also a normalized Gaussian kernel with parameter $\sqrt{\beta_{\;p_{1}}^{\;2}+\beta_{\;p_{2}}^{\;2}}$.

We have that for any kernel *w*,

C 5 $$\begin{array}{rl} w\;*\;\tilde{\;p} & =w\;*\;\;\overset{ˇ}{\;p}(t)\;e^{ikx}=\;\overset{ˇ}{\;p}(t)\int_{-\infty }^{\infty }\;e^{ik(x-y)}w(y)dy\\ & =\;\overset{ˇ}{\;p}(t)\;e^{ikx}\int_{-\infty }^{\infty }\;e^{-iky}w(y)\;dy=\hat{w}(k)\;\overset{ˇ}{\;p}(t)\;e^{ikx}. \end{array}$$

Similarly, we have $w\;*\;\tilde{s}=\hat{w}(k)\overset{ˇ}{s}(t)\;e^{ikx}$. Using this, we will calculate the Jacobian of the temporal change in coefficients $\overset{ˇ}{\;p}$ and $\overset{ˇ}{s}$ close to the steady state by substituting $\tilde{\;p}=\overset{ˇ}{\;p}\;e^{ikx}$ and $\tilde{s}=\overset{ˇ}{s}\;e^{ikx}$ into (C 2). At the homogeneous steady state *s*_0_ = *σ*(*p*_0_), *w* ∗ *p*_0_ = *p*_0_ and *w* ∗ *s*_0_ = *s*_0_.

Factoring out e^i*kx*^ and writing in matrix form, we get

C 6 $$\begin{array}{c} \left(\begin{array}{c} \overset{\dot{\vee }}{p}\\ \overset{\dot{\vee }}{s} \end{array}\right)\\ \;=\left[\begin{array}{cc} -Ds_{0}(1-s_{0})(1-{\hat{w}}_{p_{2}}(k))\; & Dp_{0}(1-{\hat{w}}_{\;p_{2}}(k))\;\cdot \\ \; & \left((1-s_{0}){\hat{w}}_{p_{1}}(k)-s_{0}\right)\\ \left(f+gs_{0})\sigma^{\prime }(p_{0}){\hat{w}}_{s}(k\right)\; & -(f+gs_{0}) \end{array}\right]\left(\begin{array}{c} \overset{\vee }{p}\\ \overset{\vee }{s} \end{array}\right). \end{array}$$

This Jacobian can be validated against the simulation to show it correctly approximates the behaviour of the system close to the steady state.

**C.1.1. Conditions for spatial instability**

Using the Jacobian from equation (5.5), also shown above in equation (C 6), we wish to prove the following:

*For Gaussian kernels, there are unstable frequencies if and only if *s*_0_(1 − *s*_0_)/(p_0_*σ*′(p_0_)) + *s*_0_ < (1 − *s*_0_). If this holds, then instability occurs in a window (0, k_c_) for some critical frequency k_c_.*

If the system is stable, then both eigenvalues of *J* will have negative real part which is the case when tr(J) and det(J)>0. We have that for the Fourier transform of a Gaussian kernel, $\hat{w}\leq 1$. Moreover, *s*_0_ ≤ 1 and so *tr*(*J*) < 0 for all frequencies *k*.

Therefore, we only need to look at the sign of det(J) which can be rearranged as

C 7 $$det(J(k))=D(1-{\hat{w}}_{\;p_{2}})(f+gs_{0})p_{0}\sigma^{\prime }(p_{0})\left(\frac{s_{0}(1-s_{0})}{p_{0}\sigma^{\prime }(p_{0})}+s_{0}{\hat{w}}_{s}-(1-s_{0}){\hat{w}}_{s}{\hat{w}}_{\;p_{1}}\right).$$

We note that det(*J*(0)) = 0 and lim_*k*→∞_ *det*(*J*(*k*)) = *D*(*f* + *gs*_0_) *s*_0_(1 − *s*_0_) > 0. Therefore, we look for a second zero of det(*J*(*k*)). If a second solution to *det*(*J*) = 0 exists at *k* = *k*_*c*_ and no third solution exists, then det(*J*) < 0 for *k* ∈ (0, *k*_*c*_] and det(*J*) > 0 for *k* ∈ (*k*_*c*_, ∞); that is, the steady state is unstable to perturbations with wavelengths *k* ∈ [0, *k*_*c*_).

In order to analyse det(*J*), we first define $F(k)=s_{0}(1-s_{0})/\left(\;p_{0}\sigma^{\prime }(p_{0})\right)+s_{0}{\hat{w}}_{s}(k)$, $G(k)={\left(1-s_{0}\right)}^{\hat{w}}{\;}_{s}{\left(k\right)}^{\hat{w}}p_{1}(k)$. For *k* > 0, *det*(*J*(*k*)) = 0 iff *F*(*k*) − *G*(*k*) = 0. *We claim that a non-zero solution to F(k) = G(k) exists if and only if *s*_0_(1 − *s*_0_)/(p_0_*σ*′(p_0_)) + *s*_0_ < 1 − *s*_0_ and that if it exists, this solution is unique*.

We will use the fact that ${\hat{w}}_{s}(k){\hat{w}}_{\;p_{1}}(k)=e^{-\pi^{2}k^{2}(1/\beta_{s}+1/\beta_{\;p_{1}})}$. From the shape of Gaussian functions, ${\hat{w}}_{s}{\hat{w}}_{\;p_{1}}<{\hat{w}}_{s}\;\forall \;k>0$. Secondly, ${\hat{w}}_{s}{\hat{w}}_{\;p_{1}}$ is steeper than ${\hat{w}}_{s}$. That is that $(d/dk)({\hat{w}}_{s}{\hat{w}}_{\;p_{1}})<(d/dk){\hat{w}}_{s}<0$.

Now assume that *s*_0_(1 − *s*_0_)/(*p*_0_*σ*′(*p*_0_)) + *s*_0_ < (1 − *s*_0_). Then *F*(0) < *G*(0). However, lim_*k*→∞_ *F*(*k*) = *s*_0_(1 − *s*_0_)/(*p*_0_*σ*′(*p*_0_)) > 0 and lim_*k*→∞_ *G*(*k*) = 0. Therefore, there must be at least one crossing point and hence a solution to *det*(*J*) = 0.

Conversely, if *s*_0_(1 − *s*_0_)/(*p*_0_*σ*′(*p*_0_)) + *s*_0_ > 1 − *s*_0_, we must look at two cases.

Case 1: *s*_0_ > (1 − *s*_0_). For all *k* > 0,

$$F(k)\geq s_{0}{\hat{w}}_{s}>(1-s_{0}){\hat{w}}_{s}>(1-s_{0}){\hat{w}}_{s}{\hat{w}}_{\;p_{1}}=G(k).$$

Therefore, F(k)≠G(k) ∀ k>0.

Case 2: *s*_0_ ≤ (1 − *s*_0_). We have that *F*(0) = *s*_0_(1 − *s*_0_)/(*p*_0_*σ*′(*p*_0_)) + *s*_0_ > 1 − *s*_0_ = *G*(0).

$$F^{\prime }(k)=s_{0}\frac{d{\hat{w}}_{s}}{dk}\geq (1-s_{0})\frac{d{\hat{w}}_{s}}{dk}\geq (1-s_{0})\frac{d({\hat{w}}_{s}{\hat{w}}_{\;p_{1}})}{dk}=G^{\prime }(k).$$

So we have that *F*′(*k*) > *G*′(*k*) and *F*(0) > *G*(0). Therefore, these two functions are never equal and hence there is no solution to *F*(*k*) = *G*(*k*). Thus a second solution to *det*(*J*) = 0 exists if and only if *s*_0_(1 − *s*_0_)/(*p*_0_*σ*′(*p*_0_)) + *s*_0_ < 1 – *s*_0_.

Finally, we must show the solution is unique. Assume that there is a solution to *F*(*k*) = *G*(*k*). This implies that *s*_0_(1 − *s*_0_)/(*p*_0_*σ*′(*p*_0_)) + *s*_0_ < 1 − *s*_0_ and hence *s*_0_ < 1 − *s*_0_. Using case 2 from above, $F^{\prime }(k)>G^{\prime }(k)\;\forall \;k>0$. As the derivatives are never equal, Rolle’s theorem shows that the solution must be unique in the range *k* > 0.

Thus we have showed that det(*J*) > 0 for sufficiently large *k*. Therefore, if there are no solutions to *F*(*k*) = *G*(*k*), then det(*J*) > 0 for all *k* > 0. A solution can occur if and only if *s*_0_(1 − *s*_0_)/(*p*_0_*σ*′(*p*_0_)) + *s*_0_ < 1 − *s*_0_ and if it exists, this solution is unique

We note that in a typical reaction–diffusion equation, Turing instabilities occur due to the interplay of the two processes (reaction and diffusion). However, this result shows that, in our model, instabilities occur only if the reaction of services to a change in population is sufficient. It is independent of the relative rates (figure 9). This independence is largely due to the conservation of population (in the first two model variations) which means that there is a zero eigenvalue at frequency *k*=0 and instabilities occur in a window [0, *k*_*c*_] rather than [*k*_1_, *k*_2_] as in a reaction–diffusion equation.

**C.1.2. Dispersion relations for other parameters**

Figure 4*a* shows dispersion relations for the default set of parameters as the total population $\bar{\;p}$ varies. Here, we show dispersion relations for the other parameters in the model illustrating how changing them modifies the predicted emergent pattern.

This gives some idea of the impact that uncertainty about parameter values may have on model predictions. Overall, pattern formation is robust and predicted across a wide range of parameters. On the other hand, the specific wavelength of predicted patterns does vary with certain key parameters as expected; parameters controlling length scales of movement ($\beta_{\;p_{1}},\beta_{\;p_{2}},\beta_{s}$) and feedback (*μ*) significantly modulate wavelengths, whereas those affecting rates of movement (*D*) and service growth (*f*, *g*) have a much weaker effect. See figure 9.

**C.2. Out-of-phase patterning requires *α*_1_ > 0 and** ${\hat{w}}_{\;p_{1}}(k)<0$

Here, we extend the linear analysis to the model with services competing for space with population (*α*_1_ > 0) and show that out of phase spatial instability is predicted only if *α*_1_ > 0 and ${\hat{w}}_{\;p_{1}}(k)<0$. This condition on the population location preference kernel corresponds to the population preferring to move to locations near to but some preferred distance from services (*a*_*p*_ > 0 in equation (6.1)).

Including competition, the linearized dynamics close to the spatially homogeneous steady state is given by

C 8 $$\begin{array}{c} \left(\begin{array}{c} \overset{\dot{\vee }}{p}\\ \overset{\dot{\vee }}{s} \end{array}\right)\\ \;=\left[\begin{array}{cc} -Ds_{0}(1-s_{0})(1-{\hat{w}}_{p_{2}}(k))\; & Dp_{0}(1-{\hat{w}}_{\;p_{2}}(k))\;\cdot \\ \; & \left((1-s_{0}){\hat{w}}_{p_{1}}(k)-s_{0}\right)\\ \left(f+gs_{0})(\sigma^{\prime }(p_{0}){\hat{w}}_{s}(k)-\alpha_{1}\right)\; & -(f+gs_{0}) \end{array}\right]\left(\begin{array}{c} \overset{\vee }{p}\\ \overset{\vee }{s} \end{array}\right). \end{array}$$

As previously, linear stability occurs when tr(J(k))<0 and det(J(k))>0. For Gaussian ${\hat{w}}_{\;p_{2}}$, the trace is negative for all *k* and therefore there are instabilities occurring for det(*J*(*k*)) < 0.

C 9 $$\begin{array}{rl} & \det(J(k))=D(1-{\hat{w}}_{\;p_{2}}(k))(f+gs_{0})\\ & \cdot (s_{0}(1-s_{0})-p_{0}(\sigma^{\prime }(p_{0}){\hat{w}}_{s}(k)-\alpha_{1})((1-s_{0}){\hat{w}}_{\;p_{1}}(k)-s_{0})) \end{array}$$

C 10 $$\det(J)\leq 0\;⟺\;s_{0}(1-s_{0})\leq p_{0}(\sigma^{\prime }(p_{0}){\hat{w}}_{s}(k)-\alpha_{1})((1-s_{0}){\hat{w}}_{\;p_{1}}(k)-s_{0})$$

C 11 $$\;⟺\;\frac{s_{0}}{p_{0}}(1-\sigma (p_{0}))+s_{0}\sigma^{\prime }(p_{0}){\hat{w}}_{s}(k)\leq (1-s_{0})(\sigma^{\prime }(p_{0}){\hat{w}}_{s}(k)-\alpha_{1}){\hat{w}}_{\;p_{1}}(k).$$

We have 1 − *σ*(*p*_0_) > 0 and, for Gaussian *w*_*s*_, ${\hat{w}}_{s}>0$. This means that the left-hand side is positive i.e. $(s_{0}/\;p_{0})(1-\sigma (p_{0}))+s_{0}\sigma^{\prime }(p_{0}){\hat{w}}_{s}>0$. For instabilities to occur the right-hand side must be therefore also be positive. The factors $\left(\sigma^{\prime }(p_{0}){\hat{w}}_{s}-\alpha_{1}\right)$ and ${\hat{w}}_{\;p_{1}}$ must at least have the same sign or else they will be negative and this condition will not hold.

If an unstable eigenvalue, *λ*, occurs, then its corresponding eigenvector will be

C 12 $$\left(\begin{array}{c} \lambda +(f+gs_{0})\\ \left(f+gs_{0})(\sigma^{\prime }(p_{0}){\hat{w}}_{s}(k)-\alpha_{1}\right) \end{array}\right).$$

As *λ* > 0 and (*f* + *gs*_0_) > 0, this vector can only be out-of-phase if $(\sigma^{\prime }(p_{0}){\hat{w}}_{s}(k)-\alpha_{1})<0$. This occurs when there is sufficient competition ($\alpha_{1}>\sigma^{\prime }(p_{0}){\hat{w}}_{s}(k))$.

As ${\hat{w}}_{\;p_{1}}(k)$ must have the same sign as $\left(\sigma^{\prime }(p_{0}){\hat{w}}_{s}(k)-\alpha_{1}\right)$ for patterning to occur at all, we must have ${\hat{w}}_{\;p_{1}}(k)<0$ as well for out-of-phase spatial instability.

**C.3. Analysis of the effect of logistic growth and competition on the stability matrix**

In this section, we show that logistic growth will tend to be stabilizing for smaller frequencies (longer wavelengths) but destabilizing for larger frequencies if there is sufficient competition. The Jacobian of the full model with competition and logistic growth from equation (7.4) is

C 13 $$\begin{array}{c} J(\;p_{0},\;s_{0},\;k)\;\\ \;\;=\left[\begin{array}{cc} -Ds_{0}(1-s_{0})(1-{\hat{w}}_{\;p_{2}}(k))-\frac{rp_{0}}{c}\; & Dp_{0}(1-{\hat{w}}_{\;p_{2}}(k))\;.\;\\ \; & \;((1-s_{0}){\hat{w}}_{\;p_{1}}(k)-s_{0})\;\\ \; & -\frac{r\alpha_{2}p_{0}}{c}\;\\ \left(\;f+gs_{0})(\sigma^{\prime }(\;p_{0}){\hat{w}}_{s}(k)-\alpha_{1}\right)\; & -(\;f+gs_{0}) \end{array}\right] \end{array}$$

Example dispersion relations can be seen in figure 10. As in previous cases, the trace will still be negative. Assuming *p*_0_, *s*_0_ remains unchanged (using equation (7.3)),

C 14 $$\begin{array}{r} \det(J)=\det(J){|}_{r=0}+\frac{rp_{0}(f+gs_{0})}{c}(1+\alpha_{2}(\sigma^{\prime }(p_{0}){\hat{w}}_{s}-\alpha_{1})). \end{array}$$

If the determinant increases such that it becomes positive for some frequency *k* (and the trace is negative), then that frequency will become a stable mode and vice versa; if the determinant becomes negative for some frequency, then that mode will become unstable. The determinant det(J) will increase if $1+\alpha_{2}(\sigma^{\prime }(p_{0}){\hat{w}}_{s}-\alpha_{1})>0$, which will occur for larger ${\hat{w}}_{s}(k)$ which occurs at smaller frequencies. Notably, the 0 frequency homogeneous solution will no longer give a zero eigenvalue but will be stable at the steady state (as shown in figure 10). This will also prevent the metastability that we saw in previous model variants.

Conversely, the determinant will decrease for some wavelengths if 1 − *α*_1_*α*_2_ < 0; that is if there is sufficient competition. This will occur for smaller values of ${\hat{w}}_{s}(k)$ which are shorter wavelength, typically out of phase, perturbations.

## Appendix D. Initial conditions for figure 5

In order to generate figure 5*a*,*b,* we initiate a two ‘bump’ and a three ‘bump’ solution with noise as can be seen in figure 11. To initialize these simulations, we begin with rectangular bumps of length 40 that give total average population $16\;000\;\text{pe}\;{\text{km}}^{-2}$. The two ‘bump’ solution has a peak of $40\;000\;\text{pe}\;{\text{km}}^{-2}$ and the three bump has $26\;000\;\text{pe}\;{\text{km}}^{-2}$. These are seeded with 100 cosine perturbations of modes 11 to 111, each of random size up to ±2% of the peak. If needed, the total population is adjusted to ensure the average is still $16\;000\;\text{pe}\;{\text{km}}^{-2}$.

## Appendix E. Fitting a logistic growth model using London data

In this section, we wish to estimate the intrinsic growth rate, *r*, in equation (7.1). To do this, we fit a simple, one dimensional, logistic growth model of the form d*p*/d*t* = *rp*(1 − (*p*/*c*)) and fit for *r*, *c* and the initial condition *p*(1801). This is done using a process of minimizing the sum of squares between the model output and the historic data. These data are London and London borough census data from 1801 to 2011 [41]. The results for London can be seen in figure 12. The values for Inner, Outer and Greater London give r=0.056,0.051 and 0.040 respectively. Therefore, *r* = 0.05 seems a reasonable estimate for the intrinsic growth rate.

## Appendix F. Varying parameters temporally

In this paper, we have made the simplifying assumption that the parameters used are static over time. This assumption is necessary in order to make the mathematical analysis of the steady states possible. However, we acknowledge that there will have been many transitions between regimes over time, especially with the advancement of technology. For example, Borchert [42] identifies five epochs from sail-wagons (*c**a* 1800) to the modern technological epoch.

The model presented here has the potential to shed further light on this in future work. For example, we show in figure 7 a simulation in which the *β* parameters vary linearly over time, starting below and ending above their default values in table 1

F 1 $$\;\begin{array}{ccc} \; & \beta_{\;p_{1}}=0.5+0.006t,\; & \beta_{s}=1+0.038t\\ \;\mathrm{and}\;\; & \beta_{\;p_{2}}=5+0.08t.\; & \end{array}$$

For simplicity, we assume there is no competition or growth; that is *r* = *a*_*p*_ = *α*_1_ = 0. This simulation shows how increasing typical travel distances over time leads to exaggerated agglomeration. Of course, growth, competition and other factors may complicate this further but this brief example shows something of the effect of socio-technological developments.
